# Clinical correlates of COVID-19 disease in psychiatric outpatients

**DOI:** 10.1192/j.eurpsy.2023.1688

**Published:** 2023-07-19

**Authors:** L. Gassab, M. jabeur, M. Ben Mbarek, B. Ben Mohamed, B. Amamou, F. Zaafrane, L. Gaha

**Affiliations:** Psychiatry, University Hospital of Monastir, Monastir, Tunisia

## Abstract

**Introduction:**

Patients suffering from psychiatric illness represent a population that is particularly vulnerable to the SARS-CoV-2 virus and to the pandemic situation due to several factors.

**Objectives:**

We aimed in our study to determine the rate of COVID-19 infection and to identify its correlated factors in outpatients of the psychiatry department of Monastir, Tunisia.

**Methods:**

This is a descriptive and analytical cross-sectional study conducted on 178 outpatients at the department of psychiatry (Monastir, Tunisia) over a period of one month (from March 2022 to April 2022). Data was collected via a questionnaire focused on two main attributes: (1) sociodemographic and clinical characteristics; (2) questions about the COVID-19 personal and family history.

**Results:**

The mean age of our population was 44.9±13.7 years. The majority of them (81.5%) had a chronic evolution of their psychiatric disorder (> 2 years) and 68.7% were hospitalized at least once in psychiatry. Psychosis was the most represented disorder with 57.3% compared to mood disorders and anxiety disorders. Among our population, thirty seven patients (21%) had a SARS-CoV-2 infection and 3.1% required hospital care. The infection by the virus of a family member was reported by 46% of cases and 2.5% had intrafamilial death due to COVID-19 disease. Patients with depressive disorder were the most affected (55%). COVID-19 infection was significantly associated with gender (p=0.02), marital status (p=0.016), diagnosis (p=0.001), treatment (p=0.02) and intrafamilial spread of the disease (p<10-3).

**Conclusions:**

Patients with psychiatric disorders experience a distinct burden of the COVID-19 disease. Awareness of the vulnerability of this population and psychiatric institutions is necessary in order to adapt mental health care planning and implement preventive measures during potential subsequent pandemics.

**Disclosure of Interest:**

None Declared

